# Supratherapeutic vitamin D with a hair nutraceutical: A case report

**DOI:** 10.1016/j.jdcr.2025.05.040

**Published:** 2025-06-30

**Authors:** Anna L. Brinks, Carli D. Needle, Caitlin Kearney, Jerry Shapiro, Kristen I. Lo Sicco

**Affiliations:** The Ronald O. Perelman Department of Dermatology, New York University Grossman School of Medicine, New York, New York

**Keywords:** alopecia, biotin, hair loss, nutraceuticals, supplements, vitamin D

## Introduction

Hair loss significantly impacts psychosocial well-being, contributing to depression, anxiety, and low self-esteem.^1^ While various medical and procedural treatments for alopecia exist, patients may opt for over-the-counter “nutraceuticals” because they are easily accessible, heavily marketed, and do not require a visit to a dermatologist. Nutraceuticals are bioactive compounds derived from food, often containing a cocktail of different vitamins, minerals, and botanical extracts.^2^ Unlike Food and Drug Administration-approved medications, supplements are not subject to rigorous premarket testing, raising questions regarding product consistency, efficacy, contamination, and understudied adverse effects. Given their often unproven efficacy, patients may also be at risk of unnecessary financial costs and further disease progression. In the randomized placebo-controlled study evaluating Nutrafol, a popular hair nutraceutical, all included subjects had self-perceived hair thinning and many had started Nutrafol within the first 6 months, making the often self-resolving telogen effluvium a possible confounder.^3^

Nutrafol's extensive ingredient list encompasses more than 23 supplements, many of which are present in amounts exceeding the recommended daily value (DV) (Table I). Ingredients include vitamin D 2500 international units (IU) (313% DV), vitamin E 3.5 mg (23% DV), biotin 3000 mcg (10,000% DV), vitamin A 1500 mcg RAE (167% DV), selenium 200 mcg (364% DV), zinc 25 mg (227% DV), and iodine 225 mcg (150% DV). These ingredient amounts reflect one serving, which is defined as 4 capsules and costs over $80 per month. Importantly, several ingredients pose overdose risk, including the fat-soluble vitamins A, D, and E as well as selenium, zinc, and iodine.^4^^,^^5^ Given the extensive ingredient list, perceived benign nature of over-the-counter products, and lack of patient education and counseling prior to nutraceutical initiation, there is potential for inadvertent overdose or inappropriate combination therapy with other supplements. Herein, we present a case of supratherapeutic vitamin D levels secondary to combination therapy with Nutrafol and supplemental vitamin D.

**Table I:** Vitamin and mineral content in best-selling hair growth nutraceuticals and women's daily multivitamins

	Vitamin D		Biotin		Vitamin A		Vitamin E		Selenium		Zinc		Iodine	
	% DV	IU	% DV	mcg	% DV	mcg	% DV	mg	% DV	mcg	% DV	mg	% DV	mcg
Nutraceuticals∗														
Nutrafol hair growth supplement	313	2,500	10,000	3000	167	1500	23	3.5	364	200	227	25	150	225
Viviscal hair growth supplement	-	-	400	120	-	-	-	-	-	-	100	11		-
Vegamour GRO gummies	-	-	16,666	5000	70	630	73	11	-	-	25	2.8	48	42
Multivitamins∗														
One a day women's multivitamin	125	1000	150	45	78	700	100	15	75	41	73	8	100	150
Centrum multivitamin for women	125	1000	133	40	117	1050	105	15.8	33	18	73	8	100	150
Nature made women's multivitamin	125	1000	100	30	83	750	150	22.5	127	70	136	15	100	150

*% DV*, Percent daily value; *IU*, International Units; *mcg*, micrograms; *mg*, milligrams.

∗ The top 3 hair growth supplements and women's daily multivitamins were determined based on the Amazon bestseller list rankings.

## Case report

A 72-year-old woman presented to the dermatology clinic for follow-up of telogen effluvium that had unmasked androgenetic alopecia. In addition to her prescribed alopecia treatments, the patient was taking 2000 IU of vitamin D daily and Nutrafol, which contains 2500 IU of vitamin D. In November 2024, laboratory testing work revealed an elevated vitamin D level of 121.8 ng/mL (normal range: 30-80 ng/mL). She did not report any clinical symptoms of vitamin D toxicity. Of note, vitamin D toxicity typically occurs when serum levels exceed 150 ng/mL, presenting with gastrointestinal distress, increased urination, confusion, lethargy, and muscle weakness.^6^

Following the laboratory results, the patient reduced her daily vitamin D supplement to 1000 IU daily but continued taking Nutrafol. In January 2025, her vitamin D level remained elevated at 115.1 ng/mL. Therefore, she was counseled to discontinue Nutrafol and continue taking just 1000 IU of vitamin D once daily. In February 2025, her vitamin D level decreased to 86.5 ng/mL, approaching the normal range.

Notably, the patient was unaware that Nutrafol contained vitamin D, leading to unintentional excessive intake. Upon detecting a supratherapeutic vitamin D level, a thorough history was necessary to determine the patient's supplement regimen. Becuase these supplements are available over the counter and routine vitamin D screening is not recommended for the general population, elevated levels and potential toxicity may go undetected in many patients.

## Discussion

While adequate vitamin and mineral levels are essential for hair health, excessive supplementation may have unintended consequences. Vitamin D toxicity, though rare, can cause severe hypercalcemia, with potentially life-threatening sequelae including renal failure and arrhythmias.^6^ Supratherapeutic levels of vitamins such as vitamin A and selenium may actually worsen alopecia, as they have been associated with hair shedding when taken in excess.^7^ Additionally, excessive iodine can contribute to thyroid dysfunction, which is a well-established cause of diffuse hair loss.^5^ Patients taking high-dose iodine supplements or consuming iodine-rich formulations should therefore be monitored for thyroid abnormalities.

Biotin, a popular supplement for hair growth that is present in Nutrafol, can also interfere with thyroid tests and cardiac biomarkers.^8^ High biotin intake has been associated with falsely suppressed thyroid-stimulating hormone levels, potentially leading to misdiagnosis of thyroid disorders.^7^ More critically, in emergency settings where an acute myocardial infarction is suspected, biotin can falsely lower cardiac troponin concentrations, potentially delaying life-saving interventions.^8^^,^^9^ Fortunately, new assays are being developed to eliminate biotin interference.^8^ Biotin is often present in hair nutraceuticals at much higher levels than recommended; Nutrafol contains 10,000% of the recommended DV (Table I).^8^ Physicians should be aware of biotin's impacts on lab results and advise patients to discontinue use before testing whenever possible.

Finally, nutraceuticals such as Nutrafol contain potentially hepatotoxic compounds, including turmeric, ashwagandha, horsetail, saw palmetto, kelp minerals, and resveratrol, with turmeric representing the highest risk (Table II).^10^ Case reports have documented liver enzyme elevations and acute liver injury following supplementation with such botanicals.^10^ A 26-year-old woman developed acute liver injury following Nutrafol use, presenting with jaundice and significantly elevated liver enzymes.^10^ Her liver function tests improved following discontinuation of the supplement, strongly implicating Nutrafol as the likely cause of her liver injury.^10^ Patients with underlying liver disease and those taking other hepatotoxic medications should avoid supplements with these ingredients.

**Table II:** Hepatotoxic agents contained in best-selling hair growth nutraceuticals

	Turmeric	Ashwagandha	Horsetail	Saw palmetto	Kelp minerals	Resveratrol
Grade of evidence for hepatotoxicity∗	B	C	C	D	E	E

Nutraceuticals						
Nutrafol hair growth supplement	Yes	Yes	Yes	Yes	Yes	Yes
Viviscal hair growth supplement	No	No	Yes	No	No	No
Vegamour GRO gummies	No	No	No	No	No	No

∗ The Drug-Induced Liver Injury Network (DILIN) uses a five-point categorization system, known as “likelihood scores,” to assess the probability of a medication causing liver injury based on case reports and published literature. Categories range from well-established links to liver injury (Category A) to drugs with no convincing evidence of liver damage (Category E).^10^

These findings emphasize the importance of patient education and physician oversight when considering hair loss supplements. While nutritional deficiencies should be corrected, excessive supplementation may lead to unintended supratherapeutic levels or adverse effects. Greater research and regulatory oversight are needed in the nutraceutical industry to ensure that supplements are both safe and effective.

## Conflicts of interest

None disclosed.
